# Supplementary material for: Matrix metalloproteinase-3 promotes arteriovenous fistula failure by regulating FAK-AKT signaling
Source: bioRxiv. 2025 Aug 30:2025.08.27.672378. Preprint. [Version 1] doi: 10.1101/2025.08.27.672378 (PMC12407871; doi:10.1101/2025.08.27.672378)

bioRxiv preprint doi: https://doi.org/10.1101/2025.08.27.672378; this version posted August 30, 2025. The copyright holder for this preprint (which was not certified by peer review) is the author/funder, who has granted bioRxiv a license to display the preprint in perpetuity. It is made available under aCC-BY 4.0 International license.

Supplementary Figure legends

bioRxiv preprint doi: https://doi.org/10.1101/2025.08.27.672378; this version posted August 30, 2025. The copyright holder for this preprint (which was not certified by peer review) is the author/funder, who has granted bioRxiv a license to display the preprint in perpetuity. It is made available under aCC-BY 4.0 International license.

Suppl. Fig. 1. Deficiency of MMP-3 does not significantly affect hemodynamic changes after AVF. 9-week-old male MMP-3-WT and MMP-3-KO mice were subjected to AVF creation. The diameter, blood flow, and velocity of the IVC were monitored by ultrasound. Shear stress was calculated based on the velocity and diameter. **A**, Representative ultrasound showed outward remodeling in the IVC after AVF. **B**, Hemodynamic data of the IVC. Quantitative data showed that MMP-3 deletion did not significantly affect the diameter, blood flow, velocity, and shear stress in the IVC after AVF. n=6. Data were analyzed using two-way ANOVA with Tukey's test for multiple comparisons for each time point. $P<0.05$ was significant. **AVF**, arteriovenous fistula; **IVC**, inferior vena cava; **Ao**, aorta.

Suppl. Fig. 2. Effect of MMP-3 deficiency in SMCs on the changes in hemodynamics after AVF. 7-week-old male SMMHC-Cre^{+}/MMP-3-flox$^{+/+}$ mice were IP injected with 75 mg/kg tamoxifen (TMX) or Oil for 5 consecutive days, followed by 2 weeks of rest, and then subjected to AVF creation. Mice were harvested after 42 days. **A**, Immunofluorescence staining showed that MMP-3 expression was largely reduced in the IVC in TMX-injected SMMHC-Cre^{+}/MMP-3-flox$^{+/+}$ mice compared with Oil-injected mice after AVF. **B**, Hemodynamic data of the IVC. Quantitative data showed that SMC-specific MMP-3 deletion did not significantly affect AVF-mediated hemodynamic changes. n=5-6. Data were analyzed using two-way ANOVA with Tukey's test for multiple comparisons for each time point. $P<0.05$ was significant. **IVC**, inferior vena cava; **Ao**, aorta; **M**, media; **N**, neointimal area; **L**, lumen; **DAPI**, nucleus.

Suppl. Table 1

qPCR primers	Direction	Primer sequence
Rat MMP-3	Forward	5’-GGACCAGGGATTAATGGAGATG-3’
	Reverse	5’-TGAGCAGCAACCAGGAATAG-3’
Rat PCNA	Forward	5’-AGCAACTTGGAATCCCAGAACAGG-3’
	Reverse	5’-AGGAGATCACCACAGCATCTCCAA-3’
Rat cyclin B1	Forward	5’-TGCACCTGCCGAAGAATATC-3’
	Reverse	5’-CACTACAGAGGTTTGGATCACC-3’
Rat cyclin D1	Forward	5’-AACTACCTGGACCGTTTCTTG-3’
	Reverse	5’-GGGAATGGTCTCCTTCATCTTAG-3’
Rat cyclin E1	Forward	5’-CTGGATGTTGGCTGCTTAGA-3’
	Reverse	5’-CACTGATAACCTGAGACCTTCTG-3’
Rat $p21^{cip1}$	Forward	5’-AAGTATGCCGTCGTCTGTTC-3’
	Reverse	5’-TCTCAGTGGCGAAGTCAAAG-3’
Rat $p27^{kip1}$	Forward	5’-ATTGGGTCTCAGGCAAACTC-3’
	Reverse	5’-TTCCTCATCCCTGGACACT-3’
Mouse MMP-3	Forward	5’-GGACCAGGGATTAATGGAGATG-3’
	Reverse	5’-TGAGCAGCAACCAGGAATAG-3’
Mouse PCNA	Forward	5’-GGCTCTCAAAGACCTCATCAA-3’
	Reverse	5’-GAGTAAGCTGTACCAAGGAGAC-3’
Human PCNA	Forward	5’-GGATACCTTGGCGCTAGTATTT-3’
	Reverse	5’-CACAGCTGTACTCCTGTTCTG-3’
Human MMP-3	Forward	5’-CCAGACTGTTGACCTCTTTGA-3’
	Reverse	5’-CGGTAGTGCCCATCATTCTT-3’
Rat GAPDH	Forward	5’-GATGCTGGTGCTGAGTATGT-3’
	Reverse	5’-GCGGAGATGATGACCCTTT-3’
Mouse GAPDH	Forward	5’-GTGGCAAAGTGGAGATTGTTG-3’
	Reverse	5’-CGTTGAATTTGCCGTGAGTG-3’
Human GAPDH	Forward	5’-GGTGTGAACCATGAGAAGTATGA-3’
	Reverse	5’-GAGTCCTTCCACGATACCAAAG-3’

Suppl. Table 2

Target antigen	Vendor or Source	Catalog #	Working concentration
Mouse anti-GAPDH	Millipore Sigma	MAB374	1:10000 (WB)
Mouse anti-SM-α-actin	Dako	M0851	1:500 (IF)
Rabbit anti-MMP-3	Abcam	ab53015	1:500 (WB); 1:50 (IF)
Mouse anti-Ki67	Thermo FIsher Scientific	14-5698-82	1:300 (IF)
Mouse anti-PCNA	Santa Cruz	sc-56	1:500 (IF)
Rabbit anti-p-AKT (Ser473)	Cell Signaling	9271S	1:500 (WB); 1:200 (IF)
Rabbit anti-AKT	Cell Signaling	9272S	1:500 (WB)
Rabbit anti-ERK 1	Santa Cruz	SC-94	1:1000 (WB)
Rabbit anti-p-ERK1/2	Cell Signaling	9101S	1:500 (WB)
Mouse anti-FAK	Invitrogen	39-6500	1:500 (WB); 1:500 (IF)
Rabbit anti-p-FAK (Tyr397)	Invitrogen	44-624G	1:500 (WB); 1:500 (IF)
Mouse anti-Fibronectin	Santa Cruz	sc-271098	1:1000 (WB)
Rabbit anti-COL1A	Santa Cruz	sc-59772	1:1000 (WB)

Suppl. Fig. 1

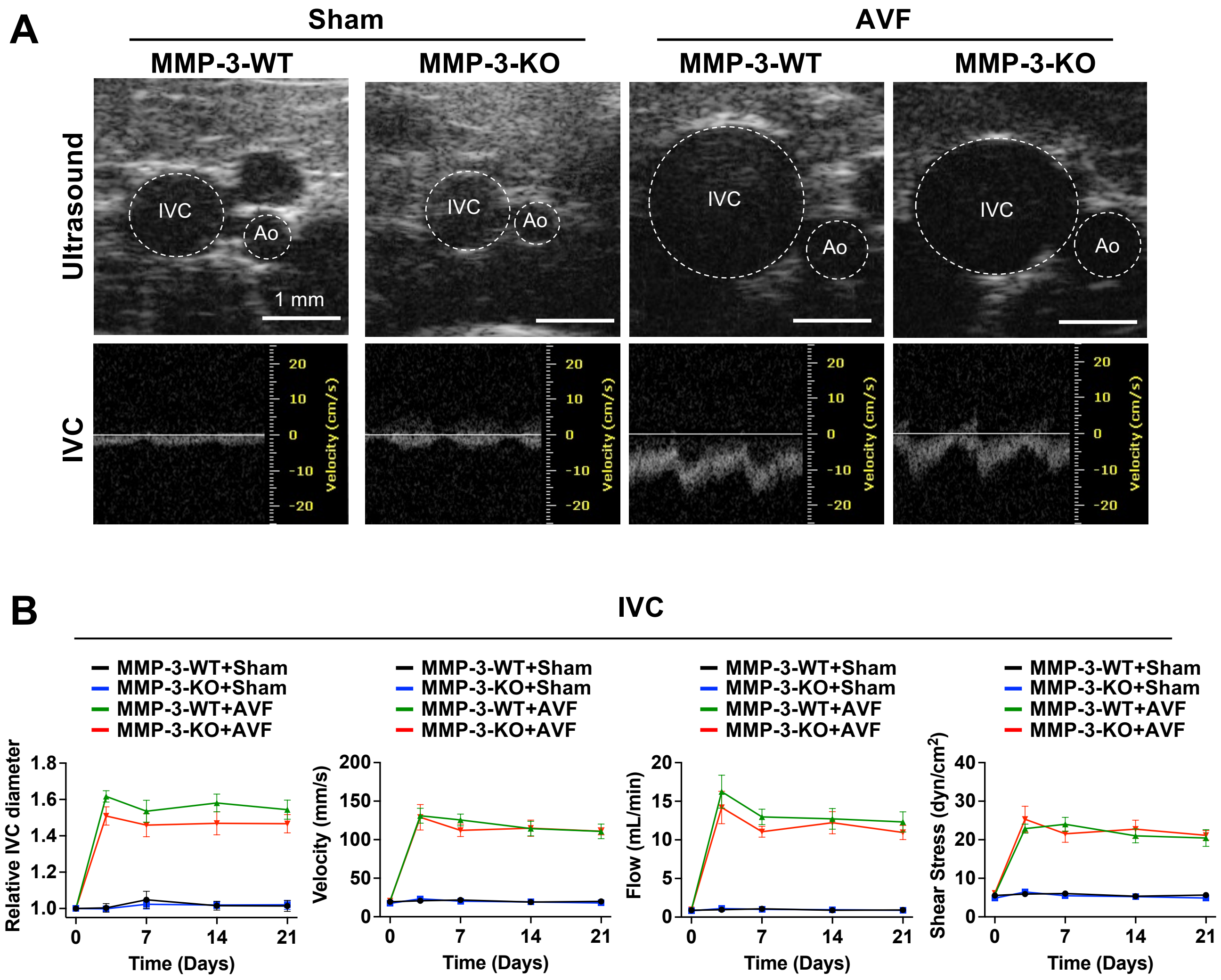
A
Sham
AVF
MMP-3-WT
MMP-3-KO
MMP-3-WT
MMP-3-KO
Ultrasound
IVC
IVC
Ao
1 mm
Velocity (cm/s)
20
10
0
-10
-20
B
IVC
MMP-3-WT+Sham
MMP-3-KO+Sham
MMP-3-WT+AVF
MMP-3-KO+AVF
Relative IVC diameter
Velocity (mm/s)
Flow (mL/min)
Shear Stress (dyn/cm²)
Time (Days)
0
7
14
21

Suppl. Fig. 2

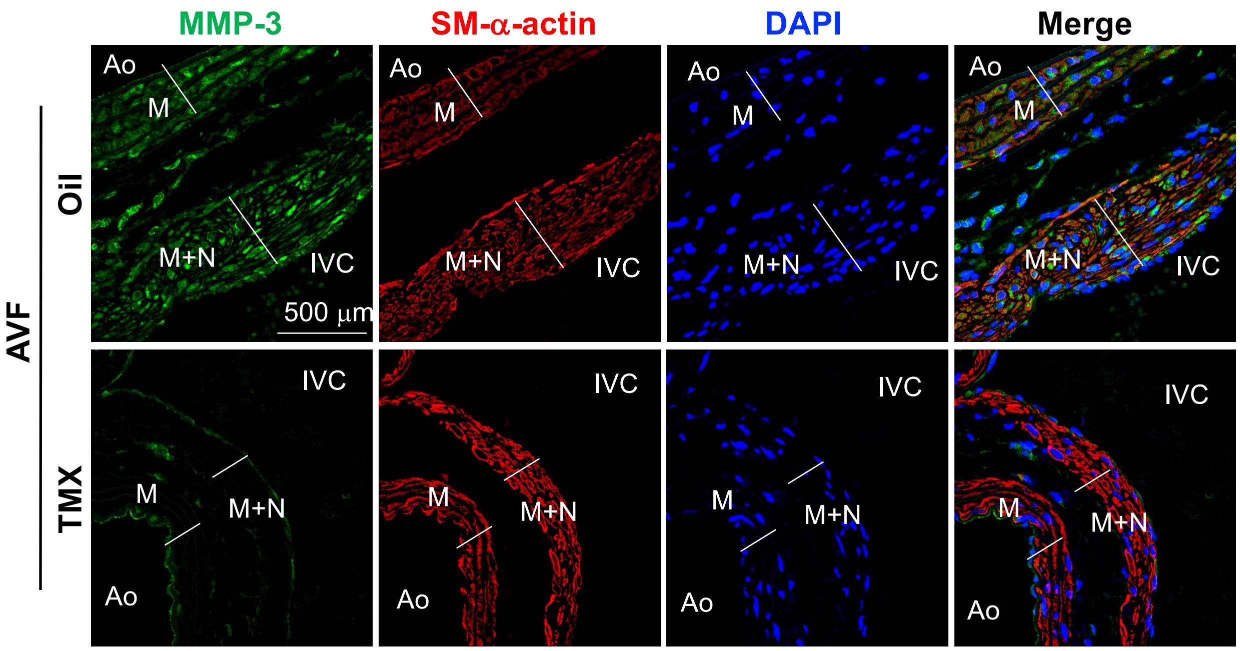

Supplement: Supplement 1 [file NIHPP2025.08.27.672378v1-supplement-1.pdf]
